# Companion Diagnostics and Predictive Biomarkers for MET-Targeted Therapy in NSCLC

**DOI:** 10.3390/cancers14092150

**Published:** 2022-04-26

**Authors:** Jan Trøst Jørgensen, Jens Mollerup

**Affiliations:** 1 Department: Medical Sciences, Dx-Rx Institute, Baunevaenget 76, 3480 Fredensborg, Denmark; 2 Pathology Division, Agilent Technologies Denmark ApS, Produktionsvej 42, 2600 Glostrup, Denmark; jens.mollerup@agilent.com

**Keywords:** MET, NSCLC, *MET* exon 14 skipping mutation, *MET* amplification, NGS, FISH, crizotinib, capmatinib, tepotinib, amivantamab

## Abstract

**Simple Summary:**

MET is a receptor tyrosine kinase encoded by the *MET* proto-oncogene that has a significant role in cancer cell progression. Several drugs targeting MET are under development for the treatment of different cancers, including non-small-cell lung cancer (NSCLC). However, until now, relatively few of these drugs have shown sufficient clinical activity and obtained regulatory approval. One of the reasons for this could be the lack of effective biomarkers to select the right patients for treatment. In a number of clinical trials, different biomarkers have been studied, but so far, *MET* exon 14 skipping mutation is the only one that has shown sufficient predictive properties. Another interesting biomarker is *MET* amplification detected by fluorescence in situ hybridization (FISH), which has shown promising results in the treatment of patients with NSCLC. Future clinical research will show whether *MET* amplification by FISH is an effective predictive biomarker for MET-targeted therapy.

**Abstract:**

Dysregulation of the MET tyrosine kinase receptor is a known oncogenic driver, and multiple genetic alterations can lead to a clinically relevant oncogenesis. Currently, a number of drugs targeting MET are under development as potential therapeutics for different cancer indications, including non-small cell lung cancer (NSCLC). However, relatively few of these drugs have shown sufficient clinical activity and obtained regulatory approval. One of the reasons for this could be the lack of effective predictive biomarkers to select the right patient populations for treatment. So far, capmatinib is the only MET-targeted drug approved with a companion diagnostic (CDx) assay, which is indicated for the treatment of metastatic NSCLC in patients having a mutation resulting in *MET* exon 14 skipping. An alternative predictive biomarker for MET therapy is *MET* amplification, which has been identified as a resistance mechanism in patients with *EGFR*-mutated NSCLC. Results obtained from different clinical trials seem to indicate that the *MET*/CEP7 ratio detected by FISH possesses the best predictive properties, likely because this method excludes *MET* amplification caused by polysomy. In this article, the concept of CDx assays will be discussed, with a focus on the currently FDA-approved MET targeted therapies for the treatment of NSCLC.

## 1. Introduction

For more than 20 years, companion diagnostics (CDx) and predictive biomarkers have had a significant impact on the development of a number of targeted hematological and oncological drugs as well as their subsequent use in the clinic. The first drug to have a CDx linked to its use was the monoclonal antibody trastuzumab (Herceptin, Roche/Genentech, Basel, Switzerland/ San Francisco, CA, USA), which was approved for the treatment of HER2-positive metastatic breast cancer together with the immunohistochemical assay (IHC) HercepTest^™^ (Dako/Agilent Technologies, Glostrup, Denmark) in 1998 [1,2]. The HercepTest^™^ assay became the first ever CDx to obtain regulatory approval by the Food and Drug Administration (FDA), and, since then, the number of drug–CDx combinations have increased considerably. By the end of 2021, this number was close to 50 [3]. For most of these targeted hematological and oncological drugs, the CDx assay plays an important role in selecting patients who are likely to respond, and, without such assays, most drugs will lose their value. 

*MET* was originally discovered as the transforming gene in a chemically transformed cell line derived from human osteosarcoma [4]. Since then, it has been established that *MET* is a proto-oncogene on chromosome 7q31, and it encodes a transmembrane receptor with intrinsic tyrosine kinase activity. The receptor tyrosine kinase is also called c-Met or hepatocyte growth factor receptor (HGFR) after its ligand, hepatocyte growth factor (HGF) [4,5,6]. Dysregulation of the MET tyrosine kinase is a known oncogenic driver; however, compared to most other proto-oncogenes, the *MET* gene is special, as different genomic states such as amplification, mutation, and rearrangement can lead to a clinically relevant oncogenesis [7]. Currently, several small-molecular inhibitors and antibody-based drugs targeting MET are under development as potential therapeutics for different cancer indications, but so far, only a few of these have obtained regulatory approval and reached the clinic [8]. One of the reasons for this might be the challenges in finding the right predictive biomarkers to guide the use of these drugs. Until now, capmatinib (Tabrecta, Novartis, Basel, Switzerland) is the only MET inhibitor that has an FDA-approved CDx linked to its use. Capmatinib can be used for the treatment of patients with metastatic non-small-cell lung cancer (NSCLC), when tumors have a mutation that leads to *MET* exon 14 skipping (*MET*ex14) [9,10]. In this article, the concept of CDx assays and predictive biomarkers will be discussed, with a focus on the current FDA-approved MET inhibitors for the treatment of NSCLC.

## 2. Companion Diagnostics

The successful development of trastuzumab for HER2-positive metastatic breast cancer opened the way for the drug-diagnostic co-development model, in which a predictive biomarker test is developed along with the drug. The use of a biomarker-guided clinical enrichment strategy often leads to an increased power of the individual clinical trial and a higher likelihood of a positive outcome [2]. For trastuzumab, this strategy was of immense importance, since without a CDx assay to enrich the clinical trial population with HER2-positive patients, the clinical development program would likely have failed [11]. A few years after the regulatory approval of trastuzumab, alternative sample size calculations for the phase III trial that led to the initial approval of trastuzumab for HER2-positive metastatic breast cancer was published by Richard Simon of the US National Cancer Institute in *Clinical Cancer Research* [12]. In this trial, an enrichment strategy was used, and here, 469 HER2-positive patients were included and randomized to receive trastuzumab plus chemotherapy or chemotherapy alone [1]. One of the alternative sample size calculations was made for an all-comers trial design, where no testing for HER2 positivity was performed, and this calculation showed that the number of patients to be included would have been 8050, to demonstrate the same statistically significant difference between the two arms, as in the original phase III trial. This corresponds to 17.2 times more patients and demonstrates the importance of the clinical enrichment trial design for the development of trastuzumab and for many other cancer drugs developed for different cancer indications over the past 20 years [3,12]. 

In 2014, the FDA issued the first guideline on In Vitro Companion Diagnostic Devices in which they officially defined a CDx assay [13]. This definition states that a CDx is an assay that provides information that is essential for the safe and effective use of a corresponding therapeutic product. In relation to this definition, it was noted that an inadequate performance of a CDx assay can have severe therapeutic consequences for the individual patient, as erroneous results could lead to the withholding of appropriate therapy or the administration of an inappropriate therapy. Consequently, the FDA classifies CDx assays as high-risk Class III devices, which requires the submission of substantial documentation for both the analytical and clinical performance before the assay can be approved and used in the clinic. Most CDx assays are developed using the prospective drug-diagnostic co-development model, so both drug and diagnostic can obtain simultaneous regulatory approval [3]. Furthermore, the use of a CDx must be included in both the labeling for the drug and the diagnostic, including the labeling of any subsequent generic equivalents of the drug. This emphasizes the importance of the CDx assay, and testing must be performed before prescribing the drug to the patient [13]. 

Several other countries worldwide, inducing the European Union (EU), have introduced similar definitions of a CDx assay as the FDA, and have tightened documentation requirements and regulatory approval procedures. In 2017, the European Parliament passed new regulations on In Vitro Diagnostic Medical Devices (IVDR) that will have a great impact on the development and use of CDx assays in European countries, as CE-IVD marking based on a self-declaration will no longer be possible [14]. The new IVDR was supposed to come into force in May 2022, but due to the extraordinary circumstances mainly caused by the COVID-19 pandemic, the European Commission has partly proposed to postpone the effective date for CDx assays to May 2026 [15]. 

## 3. MET-Targeted Therapy and NSCLC

**Figure 1:**
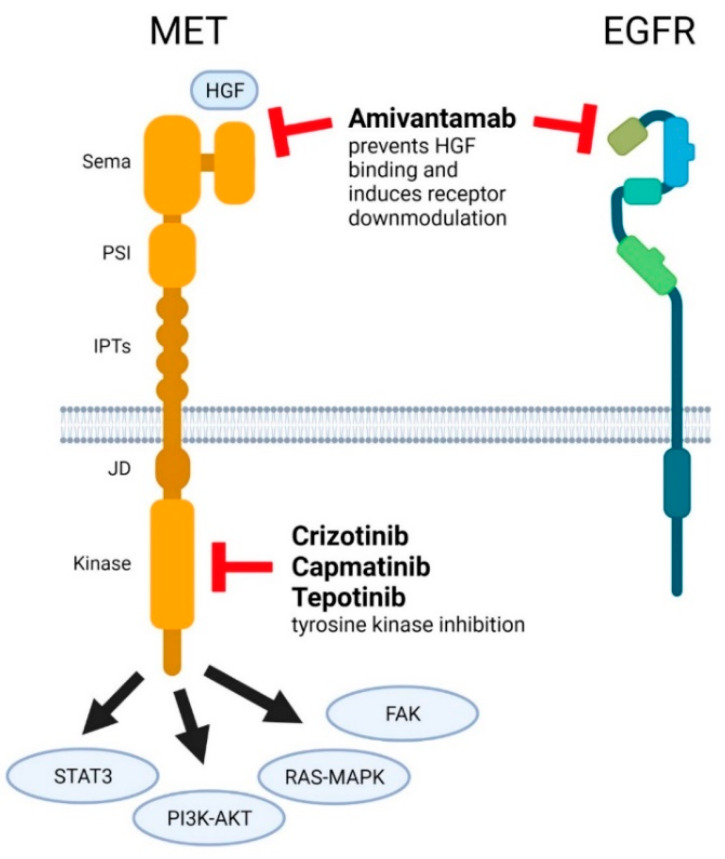
Target regions of MET inhibitors. Cartoon overview of the intra- and extracellular-domain structure of MET and the sites of inhibitor binding to MET and EGFR. Four major signaling pathways involved in MET signaling are indicated. Abbreviations: hepatocyte growth factor (HGF), Semaphorin (Sema), plexin-semaphorin-integrin (PSI), integrin-plexin-transcription factor (IPTs), juxtamembrane (JD), and epidermal growth factor receptor (EGFR).

Within different cancers, *MET* dysregulations can serve as primary drivers in promoting tumor growth, invasion, angiogenesis, and metastasis. NSCLC *MET* dysregulations in the form of *MET* amplification (*MET*amp) have also been shown to act as secondary drivers that can mediate resistance to targeted therapy for other oncogenes such as epidermal growth factor receptor (*EGFR*) mutations [7,8,16]. *MET* dysregulations have been found in 5% to 26% of NSCLC patients following treatment with an EGFR tyrosine kinase inhibitor (TKI) [17]. The first MET inhibitor to obtain FDA approval was the multi-kinase inhibitor crizotinib (Xalkori, Pfizer, New York, NY, USA), which was approved for the treatment of patients with *ALK-*rearranged NSCLC more than 10 years ago [18]. Subsequently, a few other drugs targeting MET have likewise been approved for various NSCLC indications (Figure 1). These drugs and their CDx assays are listed in Table 1 and described in more detail below. In addition, the target sites in relation to MET domains are schematically illustrated in Figure 1.

### 3.1. Crizotinib

Crizotinib is a small molecule inhibitor of receptor tyrosine kinases including ALK, MET, and ROS1, and belongs to the class Ia MET inhibitors [8,18]. Different in vitro studies have demonstrated a concentration-dependent inhibition by crizotinib of tyrosine phosphorylation mediated by ALK, ROS1, and MET in different tumor cell line-based assays. Furthermore, crizotinib has been used in vivo to show antitumor activity in mice having tumor xenografts that expressed MET or on the fusion proteins EML4-ALK or NPM-ALK [18]. 

The first indication in which crizotinib demonstrated important clinical activities was in metastatic NSCLC patients with *ALK*-rearrangement, which led to a regulatory approval of the drug by the FDA in 2011 [18,21,22]. In 2016, this indication was expanded to include NSCLC patients with *ROS1* rearrangement [18,23]. For the selection of patients for treatment with crizotinib, the FDA has approved CDx assays for the detection of both *ALK-* and *ROS1*-rearrangements [10]. Today, crizotinib must be regarded as a well-established therapy in NSCLC patients with *ALK* or *ROS1* rearrangement, but when it comes to patients with MET dysregulations, the documentation is less convincing, and a regulatory approval for this indication has not yet been granted. 

A few clinical trials have shown varying activity of crizotinib in NSCLC patients with *MET*amp or an *MET*ex14 mutation, with objective response rates (ORR) in the range of 12% to 32% depending on the type of *MET* dysregulation [24,25,26]. In these trials, the *MET*ex14 mutation was detected by next-generation sequencing (NGS) and *MET*amp by fluorescence in situ hybridization (FISH). Different cut-off values were applied with regard to the detection of *MET*amp by FISH. In one of the trials, the cut-off value was based on a *MET*/CEP7 ratio > 2.2, and in another trial, it was based on a *MET* gene copy number (GCN) ≥ 6. The results of the different reported MET trials with crizotinib are shown in Table 2. 

### 3.2. Capmatinib

Capmatinib is a small molecule kinase inhibitor that belongs to the MET-class Ib inhibitors [8]. In murine tumor xenograft models derived from human lung tumors, capmatinib has been shown to inhibit tumor growth driven by *MET*ex14 mutation or *MET*amp [8,9]. Capmatinib exerts its activity by inhibiting the MET phosphorylation triggered by the binding of HGF or by *MET*amp, as well as MET-mediated phosphorylation of the different downstream signaling proteins, which results in the impaired proliferation and survival of the MET-dependent tumor cells [9].

In a phase I trial, a possible biomarker enrichment strategy for capmatinib was investigated. Here, 55 pretreated metastatic NSCLC patients with MET dysregulation were treated with capmatinib as monotherapy [27]. Several different approaches were used to test for MET dysregulation, including overexpression as IHC2+ or IHC3+ in ≥50% of the tumor cells by IHC and *MET* GCN ≥ 5 or *MET*/CEP7 ratio ≥ 2.0 by FISH. In addition, the *MET*ex14 mutation was detected in a small subset of patient samples using NGS. Overall, for all enrolled patients, an ORR of 20% was observed. For the subgroup of patients with a *MET* GCN ≥ 6 (n = 15), an ORR of 47% was shown. For the group of patients with MET overexpression as IHC3+ (n = 37), the ORR was 27%. *MET*ex14 mutation was detected in four patients, and all responded to treatment with capmatinib. For more results from the phase I trial, please see Table 2. Based on the results from this explorative biomarker trial, it was concluded that capmatinib showed meaningful clinical activity in pretreated metastatic NSCLC patients with either *MET* GCN ≥ 6 or an *MET*ex14 mutation. When it comes to MET overexpression by IHC, this method was not considered a reliable predictive biomarker for the efficacy of capmatinib [27].

Another phase Ib/II trial also showed clinical activity of capmatinib in MET-dysregulated NSCLC patients with acquired EFGR-TKI resistance [17]. In this trial, 161 patients with MET dysregulation were selected based on *MET*amp by FISH or MET overexpression by IHC and were treated with capmatinib plus gefitinib (Iressa, AstraZeneca). For the phase Ib part, the patient selection criteria were either *MET* GCN ≥ 5 and/or a *MET*/CEP7 ratio ≥ 2.0 or MET overexpression as IHC2+ or IHC3+ in ≥ 50% of the tumor cells. For the phase II part, the selection criteria were initially defined as *MET* GCN ≥ 5 or MET IHC2+ or IHC3+ overexpression in ≥ 50% of tumor cells. However, in a protocol amendment, these criteria were revised to MET IHC2+ or IHC3+ plus *MET* GCN ≥ 5; these were subsequently changed once more to MET IHC3+ or *MET* GCN ≥ 4. Across the phase Ib/II trial and the different MET or *MET* selection criteria, the observed ORR was 27%. In a post-hoc subgroup analysis, an ORR of 47% was shown for phase II patients with *MET* GCN > 6 (n = 36). For patients with MET IHC3+ (n = 78), the ORR was 32%, and for the MET IHC2+ group (n = 16), the ORR was 19%. For more results from the phase Ib/II trial, please see Table 2. Overall, the post-hoc subgroup analysis showed that *MET* FISH using a cut-off value of *MET* GCN > 6 was more accurate compared to MET IHC in predicting the response in NSCLC patients receiving a combined treatment of capmatinib and gefitinib [17]. 

In a prospective, open-labeled, multiple-cohort phase 2 trial (GENOMETRY mono-1), capmatinib was further investigated in metastatic NSCLC patients with an *MET*ex14 mutation or *MET*amp [28]. The patients in this trial were assigned to different cohorts based on *MET* status and previous treatment. *MET*ex14 mutation was initially determined by a qualitative real-time reverse-transcription PCR (RT-PCR) assay. Detection of *MET*amp, as GCN, was initially determined by FISH. Subsequently, the *MET*ex14 mutation and *MET* GCN were retrospectively retested based on the baseline tissue samples from the trial using the NGS FoundationOne CDx assay (Foundation Medicine). A total of 364 patients were assigned to different study cohorts. For the group with the *MET*ex14 mutation, an ORR of 68% was obtained in the treatment-naïve patients (n = 28) compared to the previously treated patients (n = 69) with an ORR of 41%. In the patients with *MET*amp and a GCN ≥ 10, the ORR was 40% in the treatment-naïve (n = 15) and 29% in those previously treated (n = 69). For the patient cohorts previously treated with *MET* GCN < 10, the activity of capmatinib seemed to be limited, with ORR in the range of 7 to 12% [28]. For more results from the phase II trial, please see Table 2. In 2020, based on data from 97 patients with a *MET*ex14 mutation in the GENOMETRY mono-1 trial, capmatinib obtained FDA approval [9], and, along with this approval, the NGS FoundationOne CDx assay was approved as the CDx for the detection of the *MET*ex14 mutation in NSCLC patients who may benefit from treatment with capmatinib [10,31]. 

### 3.3. Tepotinib

Tepotinib (Tepmetko, Merck/EMD Serono; Darmstadt, Germany/Rockland, MA, USA) is a small molecule-class Ib inhibitor that targets MET by inhibiting HGF-dependent and -independent MET phosphorylation as well as the MET-dependent downstream signaling pathways [8]. In vitro and in vivo studies have shown that tepotinib inhibited the growth of MET-dysregulated tumor cells, and mice implanted with tumor cells expressing oncogenic active MET had a reduced formation of metastases [19].

In a phase Ib/II trial, the clinical activity of tepotinib was demonstrated in metastatic NSCLC patients with MET dysregulation who had developed a resistance to EGFR-TKI [29]. A total of 73 patients, 18 in the phase Ib part and 55 in the phase II part, were enrolled. In the phase II part, the patients were randomized to receive either tepotinib plus gefitinib or chemotherapy. MET dysregulation was defined as MET IHC2+ or IHC3+ by IHC or *MET*amp as GCN ≥ 5 or *MET*/CEP7 ratio ≥ 2.0 by FISH. In the phase 1b part, four of the seven patients with MET IHC3+ responded, which was similar for four of the six patients with *MET*amp. In the phase 2 part of the trial, 13 of the 19 MET IHC3+ patients responded (ORR 68%), which was similar to the *MET*amp patients, with 8 of 12 patients responding (ORR 67%). For the chemotherapy group, the ORR was 33% for MET IHC3+ and 43% for *MET*amp patients [29]. Based on the results of this trial, it was concluded that the combination of tepotinib and gefitinib showed similar activity in MET IHC3+ and *MET*amp patients. 

In another phase II trial (VISION), tepotinib was investigated in patients with metastatic NSCLC who harbored a *MET*ex14 mutation [30]. Furthermore, the response to tepotinib was analyzed according to whether the presence of the *MET*ex14 mutation was detected from a tissue biopsy or from plasma as circulating tumor DNA (ctDNA). For the tumor tissue biopsies, the *MET*ex14 mutation was assessed using the NGS Oncomine Focus Assay (Thermo Fisher Scientific, Waltham, MA, USA), and for the liquid biopsy, the ctDNA was analyzed using another NGS assay, the Guardant360 (Guardant Health, Redwood City, MA, USA). Testing by both biopsy methods was not a requirement for inclusion in the trial. A total of 152 patients were enrolled in the trial, and in the combined biopsy group (n = 99), the ORR was 46%. For the 66 patients with liquid biopsies, the ORR was 48%, and it was 50% for the 60 patients with tissue biopsies. In 2021, based on clinical data from the VISION trial, tepotinib obtained FDA approval for the treatment of patients with metastatic NSCLC harboring the *MET*ex14 mutation [19]. Somewhat surprisingly, tepotinib was approved without the Oncomine Focus Assay and/or the Guardant360 as CDx for the detection of *MET*ex14 mutations. The full prescribing information for tepotinib emphasizes that an FDA-approved test for the detection of *MET*ex14 mutations in NSCLC for selecting patients for treatment is not available. Furthermore, it is stated that testing for the presence of *MET*ex14 mutations in plasma specimens is recommended only in patients in whom a tumor biopsy cannot be obtained. If a *MET*ex14 mutation is not detected in a plasma specimen, the possibility of a tumor biopsy should be reconsidered [19]. Information related to the use of liquid biopsies is the same as the FDA has given for other CDx assays using similar technologies [10]. The reason for this is that a negative result from a liquid biopsy does not exclude a potential oncogenic driver (here, the *MET*ex14 mutation), because some tumors do not shed a sufficient amount of DNA into the circulation to be detected by this method [32] 

### 3.4. Amivantamab

Amivantamab (Rybrevant, Jansen Biotech, Horsham, PA, USA) is a bispecific antibody targeting the EGFR and MET [33]. In vitro and in vivo studies have shown that amivantamab is able to disrupt the EGFR and MET signaling functions by ligand blocking and receptor degradation. Furthermore, amivantamab has the ability to induce trogocytosis and engage immune effector cells to eliminate EGFR and MET-presenting tumor cells through antibody-dependent cellular cytotoxicity [20,33]. 

In a multicohort open-labeled phase I trial (CHRYSALIS) in patients with metastatic NSCLC, amivantamab was investigated in different molecular-defined subgroups, including patients with *EGFR* exon 20 insertion mutation, *MET*ex14 mutation, and *MET*amp. So far, only the results from the *EGFR* exon 20 insertion mutation cohort (n = 81) have been reported, and here, amivantamab given as monotherapy showed an ORR of 40% [34]. The clinical outcome data from this cohort of the CHRYSALIS trial led to an FDA approval of amivantamab for the treatment of adult patients with locally advanced NSCLC with the *EGFR* exon 20 insertion mutation, whose disease has progressed on or after platinum-based chemotherapy [20]. Together with the approval of amivantamab, the Guardant360 assay was approved as CDx for the detection of *EGFR* exon 20 insertions [10]. However, it will be interesting to study the results from the other cohorts of the trial, patients with the *MET*ex14 mutation and *MET*amp, and to see if they can support an expansion of the current indication for amivantamab. 

## 4. Companion Diagnostics and Predictive Biomarkers for MET-Targeted Therapy 

Taking into consideration the relatively large number of MET inhibitors that have been or are under development for different indications, it is disappointing to see that only a small proportion have obtained regulatory approval and subsequently reached the clinic. Besides being investigated for treatment of NSCLC, MET inhibitors are under clinical development for different indications, such as gastric and gastroesophageal cancer, hepatocellular carcinoma, and renal cell carcinoma [7,8,35]. These investigational drugs cover both small molecule inhibitors, mono- and bispecific antibodies, as well as antibody–drug conjugates targeting MET [35,36,37]. 

One of the reasons for the relatively low success rate of MET-targeted therapy in NSCLC might be the lack of predictive biomarkers with sufficient accuracy. For the FDA-approved drugs, Table 2 lists the biomarkers that were used to select patients for MET-targeted therapy in different clinical trials. These biomarkers include overexpression by IHC, GCN, and *MET*/CEP7 ratios by FISH, GCN by NGS, and *MET*ex14 mutation by NGS. The use of MET overexpression, either MET IHC2+ or IHC3+, has given inconsistent results, and the data in Table 2 shows ORR ranging from 14% to 68% depending on the level of overexpression, with the best response obtained in patients with MET IHC3+ tumors [17,27,29]. 

The data for *MET*amp in Table 2 also shows a variation with ORR ranging from 16% to 67% for patients with *MET* GCN > 6 or a *MET*/CEP7 ratio ≥ 2.0. For a *MET*/CEP7 ratio > 2.0, the ORR following treatment with MET-targeted therapy ranges from 33% to 67%, whereas *MET* GCN > 6 predicts ORR outcomes in the range of 16% to 67%. *MET* gene copy number gains can occur through both polysomy and amplification, but it seems that true amplification is more likely to lead to oncogenic addiction, which might explain the possibly better predictive properties of *MET*/CEP7 over *MET* GCN [7,16]. NGS was used as a method for the detection of *MET*amp in patients with NSCLC, but results from several comparative studies with FISH have shown poor reliability in detecting the various levels of *MET*amp [38,39,40]. Based on the results from one of these studies comparing clinical outcome data, the authors concluded that *MET*amp identified by FISH remains the optimal biomarker to identify suitable candidates for MET-TKI therapy [39]. One possible problem in relation to the use of NGS-based assays is likely the lack of control for CEP7, whereby a detected increase in the *MET* gene copy number could be the result of polysomy rather than a true *MET*amp [16].

For NSCLC patients with identified oncogenic drivers, the use of targeted therapy has significantly improved treatment outcome, with high response rates and improved progression-free survival. However, resistance to this type of therapy will be developed sooner or later, and here, *MET*amp seems to play a central role [7,8,16]. For treatment with EGFR inhibitors, *MET*amp has been established as a mechanism of acquired resistance, and evidence is accumulating that this could also occur in NSCLC with targeted therapies related to *ALK-*, *RET-*, and *ROS1-*rearrangements. For patients with *MET*amp who have developed resistance to EGFR inhibitors, the combination with a MET inhibitor seems to overcome the resistance [16,17,29]. In this perspective, it is important to clarify whether NGS can be used as a reliable platform for the detection of *MET*amp in patients with NSCLC, or whether the recommendation should be to use a FISH assay going forward.

In Table 2, the data for *MET*ex14 mutation detected by NGS also shows variability regarding the ORR observed. However, if data originate from larger populations, it seems to show a higher degree of consistency with ORR in the range of 32% to 64% [26,28,30]. In general, when comparing the data in Table 2, it is important to have in mind that these data originate from different patient populations and line of therapy as well as different MET-targeted drugs. Furthermore, several of the patient populations listed are relatively small, which is why one should be very careful not to draw firm conclusions based on the presented data. 

So far, the FoundationOne CDx is the only assay linked to a MET inhibitor that has obtained FDA approval [10,31]. This CDx assay is used with capmatinib, and the clinical validation with respect to the detection of the *MET*ex14 mutation was performed based on samples and clinical data from the GENOMETRY mono-1 trial [28]. Here, a clinical bridging study was performed to show both analytical and clinical agreement between the enrollment assay and the FoundationOne CDx assay. In the GENOMETRY mono-1 trial, the patients were enrolled based on test results from a *MET*ex14 mutation RT-PCR assay. To establish concordance between the two assays, both cohorts of treatment-naïve and previously treated patients were analyzed. Based on the assay results, positive percent agreement (PPA), negative percent agreement (NPA), and overall agreement (OPA) were calculated. For the previously treated group of patients, PPA was 96.8%, NPA was 100%, and OPA was 99.1%. For the group of treatment-naïve patients, PPA, NPA, and OPA were all 100% and, thereby, a complete concordance between the two assays was achieved and likewise with respect to the clinical outcome [31]. 

MET-targeted therapy has also been investigated outside NSCLC, and here, it is more or less the same type of predictive biomarkers that have been used for patient selection. In a phase II trial, the experimental small molecule MET inhibitor AMG 337 was investigated in patients with gastric/gastroesophageal junction/esophageal adenocarcinoma, and a FISH assay with *MET*/CEP7 ≥ 2.0 as a cut-off was used [41,42]. Furthermore, the small molecular inhibitors tivantinib and tepotinib have been investigated in patients with hepatocellular carcinoma using MET IHC2+ and IHC3+ overexpression for enrollment in the clinical trials [43,44]. Despite these ongoing development activities, none of the different MET inhibitors have obtained regulatory approval for indications other than NSCLC so far.

## 5. Conclusions

Despite intensive research and development efforts, relatively few MET inhibitors have shown sufficient clinical activity. One of the reasons for this could be the lack of effective predictive biomarkers to select the right patient population for treatment. So far, capmatinib is the only MET inhibitor that has been approved with a CDx assay. In 2020, capmatinib obtained FDA approval for the treatment of patients with metastatic NSCLC whose tumors harbor a *MET*ex14 mutation. Likewise, in different clinical trials, the *MET*ex14 mutation has also shown predictive properties for drugs such as tepotinib and crizotinib in patients with metastatic NSCLC. Another candidate biomarker is *MET* amplification, which plays an important role in the development of achieved resistance to EGFR inhibitors. Results obtained from different clinical trials indicate that determination of the *MET*/CEP7 ratio by FISH possesses the best predictive property, likely because this approach excludes *MET* amplification caused by polysomy. However, further clinical research will have to show whether *MET*/CEP7 by FISH is an effective predictive biomarker for MET-targeted therapy. 

## Figures and Tables

**Table 1:** FDA-approved MET targeted drugs and their CDx assays. Only capmatinib and tepotinib are approved for a MET-specific indication [9,10,18,19,20].

Drug	Drug Class	Approved Indication(s)	FDA Approved CDx Assay(s)	
Crizotinib	Small molecule inhibitor	Treatment of patients with metastatic NSCLC whose tumors are ALK or ROS1-positive as detected by an FDA-approved test	ALK	FoundationOne CDx VENTANA ALK (D5F3) CDx Assay Vysis ALK Break Apart FISH Probe Kit ROS1
			ROS1	Oncomine Dx Target Test
			MET	No approved CDx available
Capmatinib	Small molecule inhibitor	Treatment of adult patients with NSCLC whose tumors have a mutation that leads to *MET* exon 14 skipping as detected by an FDA-approved test	MET	FoundationOne CDx
Tepotinib	Small molecule inhibitor	Treatment of adult patients with metastatic NSCLC harboring *MET* exon 14 skipping alterations	MET	No approved CDx available
Amivantamab	Bispecific antibody	Treatment of adult patients with locally advanced or metastatic NSCLC with *EGFR* exon 20 insertion mutations, as detected by an FDA-approved test, whose disease has progressed on or after platinum-based chemotherapy	*EGFR* exon 20 insertion	Guardant360^®^ CDx
			*MET*	No approved CDx available

CDx = Companion Diagnostic; ALK = Anaplastic Lymphoma Kinase; ROS1 = ROS proto-oncogene 1; MET = Mesenchymal Epithelial Transition Factor; EGFR = Epidermal Growth Factor Receptor; *MET*ex14 = *MET* exon 14 skipping mutation; NSCLC = Non-Small Cell Lung Cancer; FDA = Food and Drug Administration.

**Table 2:** Predictive Biomarkers and Companion Diagnostics for the FDA-approved MET Targeted Drugs in NSCLC.

Drug	Publication [Reference]	Method	Biomarker/CDx	N	Objective Response Rate
Crizotinib	Moro-Sibilot D et al. [24]	FISH NGS	*MET* GCN ≥ 6	25	16%
			*MET*ex14	25	12%
	Landi L et al. [25]	FISH NGS	*MET*/CEP7 > 2.2	16	31%
			*MET*ex14	10	20%
	Drilon A et al. [26]	NGS	*MET*ex14	65	32%
Capmatinib	Schuler M et al. [27]	FISH	*MET* GCN < 4	17	6%
			4 ≤ *MET* GCN < 6	12	25%
			*MET* GCN ≥ 6	15	47%
			*MET*/CEP7 ≥ 2.0	9	44%
			*MET*/CEP7 < 2.0	32	22%
		IHC	MET IHC2+	14	14%
			MET IHC3+	37	27%
	Wu YL et al. [17]	FISH	*MET* GCN < 4	41	12%
			4 ≤ *MET* GCN < 6	18	22%
			*MET* GCN ≥ 6	36	47%
		IHC	MET IHC2+	16	19%
			MET IHC3+	78	32%
	Wolf J et al. [28]	NGS	*MET*ex14	69 (Previous treated)	41%
			*MET*ex14	28 (Treatment naïve)	64%
		NGS	*MET* GCN < 4	30 (Previous treated)	7%
			*MET* GCN 4 or 5	54 (Previous treated)	9%
			*MET* GCN 6–9	42 (Previous treated)	12%
			*MET* GCN ≥ 10	69 (Previous treated)	28%
			*MET* GCN ≥ 10	15 (Treatment naïve)	40%
Tepotinib	Wu YL et al. [29]	IHC	MET IHC3+	19	68%
		FISH	*MET*/CEP7 ≥ 2.0	12	67%
	Paik PK et al. [30]	NGS	*MET*ex14	99	46%

CDx = Companion Diagnostic; FISH = Fluorescence In Situ Hybridization; NGS = Next-Generation Sequencing; IHC = Immunohistochemistry; MET = Mesenchymal Epithelial Transition Factor; GCN = Gene Copy Number; *MET*ex14 = *MET* exon 14 skipping mutation; CEP7 = Centromere 7.
